# A TagSNP in *SIRT1* Gene Confers Susceptibility to Myocardial Infarction in a Chinese Han Population

**DOI:** 10.1371/journal.pone.0115339

**Published:** 2015-02-23

**Authors:** Jie Cheng, Miook Cho, Jin-ming Cen, Meng-yun Cai, Shun Xu, Ze-wei Ma, Xinguang Liu, Xi-li Yang, Can Chen, Yousin Suh, Xing-dong Xiong

**Affiliations:** 1 Institute of Aging Research, Guangdong Medical College, Dongguan, P.R. China; 2 Guangdong Provincial Key Laboratory of Medical Molecular Diagnostics, Dongguan, P.R. China; 3 Institute of Biochemistry & Molecular Biology, Guangdong Medical College, Zhanjiang, P.R. China; 4 Department of Genetics, Albert Einstein College of Medicine, Bronx, New York, United States of America; 5 Department of Cardiovascular Disease, The First People’s Hospital of Foshan, Foshan, P.R. China; 6 Department of Cardiovascular Disease, The Affiliated Hospital of Guangdong Medical College, Zhanjiang, P.R. China; 7 Department of Medicine, Albert Einstein College of Medicine, Bronx, New York 10461, United States of America; University of Texas MD Anderson Cancer Center, UNITED STATES

## Abstract

*SIRT1* exerts protective effects against endothelial cells dysfunction, inflammation and atherosclerosis, indicating an important role on myocardial infarction (MI) pathogenesis. Nonetheless, the effects of *SIRT1* variants on MI risk remain poorly understood. Here we aimed to investigate the influence of *SIRT1* polymorphisms on individual susceptibility to MI. Genotyping of three tagSNPs (rs7069102, rs3818292 and rs4746720) in *SIRT1* gene was performed in a Chinese Han population, consisting of 287 MI cases and 654 control subjects. In a logistic regression analysis, we found that G allele of rs7069102 had increased MI risk with odds ratio (OR) of 1.57 [95% confidence interval (CI) = 1.15–2.16, Bonferroni corrected *P* (*P_c_*) = 0.015] after adjustment for conventional risk factors compared to C allele. Similarly, the combined CG/GG genotypes was associated with the increased MI risk (OR = 1.64, 95% CI = 1.14–2.35, *P_c_* = 0.021) compared to the CC genotype. Further stratified analysis revealed a more significant association with MI risk among younger subjects (≤ 55 years old). Consistent with these results, the haplotype rs7069102G-rs3818292A-rs4746720T containing the rs7069102 G allele was also associated with the increased MI risk (OR = 1.41, 95% CI = 1.09–1.84, *P_c_* = 0.040). However, we did not detect any association of rs3818292 and rs4746720 with MI risk. Our study provides the first evidence that the tagSNP rs7069102 and haplotype rs7069102G-rs3818292A-rs4746720T in *SIRT1* gene confer susceptibility to MI in the Chinese Han population.

## Introduction

Myocardial infarction (MI) is the world’s leading cause of morbidity and mortality, with the World Bank estimating that the number of individuals with MI in China will increase to 23 million by 2030 [1,2]. Lots of risk factors have been reported to contribute to the pathogenesis of MI, including smoking, alcohol intake, diabetes, hypertension, hypercholesterolemia, obesity, physical inactivity and psychosocial situation [3,4]. Recently, a growing body of studies have focused on associations of polymorphic variants in candidate genes with the risk of MI, providing evidence that host genetic variations exert critical roles on the pathogenesis of MI in addition to the above risk factors [5–7].

Mammalian *Sirtuin1* (*SIRT1*), the closest homolog of yeast silent information regulator 2 (Sir2), functions as a NAD^+^-dependent histone deacetylase [8,9], which is abundantly expressed in the vasculature [10]. Accumulating evidence has indicated that *SIRT1* played an important role in protection against vascular aging and age-related vascular diseases [10], including inhibiting neointima formation [11] and protecting against atherosclerosis [12,13]. Reduced *SIRT1* expression facilitated the occurrence of senescence in endothelial cells [14–16]. In contrast, overexpression or activation of *SIRT1* promoted endothelial function and suppressed vascular inflammation by mediating NAD^+^-dependent deacetylation of intracellular protein targets [17–20]. Ota *et al*. [21] found that SIRT1 inhibition increased p53 acetylation and caused a stress-induced premature senescence (SIPS)-like phenotype in endothelial cells, and *vice versa*, *SIRT1* overexpression reversed the SIPS induced by oxidative stress. Besides, *SIRT1* activation can inhibit vascular smooth muscle cell (VSMC) hypertrophy, which has been considered one of the critical contributors to atherosclerosis. Several studies had found that resveratrol was an activator of *SIRT1* [22–25]. Resveratrol protects human endothelial cells from H_2_O_2_-induced oxidative stress and senescence *via SIRT1* activation [26]. More directly, impaired cardiac SIRT1 activity plays a critical role in the observed increase in susceptibility of the aged heart to I/R injury. SIRT1 agonist can restore this aging-related loss of cardioprotection [27]. Therefore, *SIRT1* may play a critical role in the pathophysiology of MI.

Recent genome-wide association studies (GWAS) have identified various polymorphisms that confer susceptibility to MI or early-onset MI [7,28–30]. Moreover, considering that *SIRT1* gene polymorphisms can affect the protein expression in cardiovascular diseases [31], we speculated that the polymorphisms in *SIRT1* gene might have an impact on the susceptibility to MI as well. Many single nucleotide polymorphisms (SNPs) show correlated genotypes, or linkage disequilibrium (LD), suggesting that only a subset of SNPs (known as tagging SNPs, or tagSNPs) need to be genotyped for disease association studies. Therefore, we herein conducted a case-control study to elucidate the association of three *SIRT1* tagSNPs, namely rs7069102, rs3818292 and rs4746720, with the risk of MI. Our analysis revealed that the G allele of tagSNP rs7069102 and haplotype rs7069102G-rs3818292A-rs4746720T conferred susceptibility to MI in the Chinese Han population.

## Materials and Methods

### Study subjects

A total of 287 MI patients were recruited from the First People’s Hospital of Foshan (Foshan, China) and the Affiliated Hospital of Guangdong Medical College (Zhanjiang, China) from March 2011 to February 2013. All the MI patients were newly diagnosed and previously untreated. The diagnosis of MI was based on clinical symptoms and typical electrocardiographic changes, and on increases in the serum cardiac markers, such as creatinine kinase, aspartate aminotransferase, lactate dehydrogenase and troponin T. The diagnosis was confirmed by the identification of the responsible stenosis in any of the major coronary arteries or in the left main trunk by coronary angiography. A total of 654 control subjects were consecutively recruited from the participating hospitals for regular physical examinations during the same period when MI patients were recruited. The unaffected controls were judged to be free of MI by questionnaires, medical history, clinical examination and electrocardiography. Individuals with congestive heart failure, peripheral vascular disease, rheumatic heart disease, pulmonary heart disease, chronic kidney, hepatic disease, or any malignancy were excluded from the study.

All study subjects were genetically unrelated and self-reported ethnically Han Chinese. Each subject was interviewed after written informed consent was obtained, and a structured questionnaire was administered by interviewers at the enrollment to collect information on demographic data and risk factors related to MI. Meanwhile, we consulted each subject for the genetic relatedness information and excluded the subjects related to the individuals who had enrolled the study. The diagnosis of hypertension was established if patients were on antihypertensive medication or if the mean of 3 measurements of systolic blood pressure (SBP) ≥140 mm Hg or diastolic blood pressure (DBP) ≥ 90 mm Hg, respectively. Diabetes mellitus was defined as fasting blood glucose ≥7.0 mmol/L or use of antidiabetic drug therapy. Hyperlipidemia was defined as serum total cholesterol (TC) concentration >5.72 mmol/L or triglyceride (TG) concentration >1.70 mmol/L or use of lipid-lowering therapy. Individuals that smoked once a day for over 1 year were defined as smokers. The study was approved by the Medical Ethics Committee of the First People’s Hospital of Foshan and the Affiliated Hospital of Guangdong Medical College.

### Analysis of biochemical parameters

An approximately 2 ml venous blood sample was drawn from each subject into tubes containing EDTA after an overnight fast. The blood sample was centrifuged at 2000×g for 15 min immediately after collection and stored at -80°C until analysis. The levels of plasma total cholesterol (TC), triglyceride (TG), high density lipoprotein cholesterol (HDLC), and low density lipoprotein cholesterol (LDLC) were measured enzymatically using a chemistry analyzer (Olympus, Japan). Glucose was analyzed by the glucose oxidase method with an Abbott V/P Analyzer (Abbott Laboratories, USA).

### DNA extraction

Genomic DNA was extracted from peripheral whole blood by TIANamp blood DNA extraction kit (TianGen Biotech, Beijing, China) according to the manufacturer’s instructions. All DNA samples were dissolved in water and stored at -20°C until use.

### TagSNP selection and genotyping

The Chinese Han population’s SNP data of *SIRT1* gene (33.72kb, 9 exons) was downloaded from the HapMap data release 27 (http://www.hapmap.org). We analyzed these data by using Haploview software version 4.2 [32]. A minor allele frequency (MAF) > 0.05 and a linkage disequilibrium measure (r^2^) > 0.8 were prerequisites for tagSNPs selection. We then got three tagSNPs, including rs7069102, rs3818292 and rs4746720. rs7069102 is located in intron 4 and rs3818292 is located in intron 5 of the *SIRT1* gene while rs4746720 lies in the 3′ untranslated region (UTR) (S1A Fig.). According to the SHEsis platform[33], the r^2^ between rs7069102 and rs3818292 was 0.065, between rs7069102 and rs4746720 was 0.134, between rs3818292 and rs4746720 was 0.280, indicating that they did not exist in linkage disequilibrium with each other. These three tagSNPs would capture the information of 18 known *SIRT1* SNPs with a MAF > 0.05 (S1B Fig.). The r^2^ information for these tagSNPs and alleles captured accordingly was shown in the S1 Table. The potential functions of the SNPs were predicted by online webserver (http://snpinfo.niehs.nih.gov/snpinfo/snpfunc.htm). Since haplotype analysis would reduce the dimensionality8 of association tests and increase the statistical power, the haplotypic blocks of the three tagSNPs were also estimated by the Haploview software version 4.2. Then the haplotype analysis was performed with the SHEsis platform.

Genomic DNA was genotyped by polymerase chain reaction-ligase detection reaction (PCR-LDR) method (Shanghai Biowing Applied Biotechnology Company). The sequence of primers and probes are summarized in S2 Table. The PCR was carried out on the ABI 9600 (Applied Biosystems, USA) in a total volume of 20 μl including 50 ng genomic DNA, 1 × PCR buffer, 3 mM MgCl_2_, 2 mM dNTPs, 0.5 μM each primer, and 1 U hot-start Taq DNA polymerase (Qiagen). Cycling parameters were as follows: 95°C for 15 min; 35 cycles at 94°C for 30 sec, 65°C for 1 min and 30 sec, 72°C for 1 min and 30 sec; and a final extension step at 72°C for 7 min. The ligation reaction for each PCR product was carried out with a final volume of 10 μl containing 1 μl 1 × ligation buffer, 1 μl of PCR product, 12.5 pmol of each discriminating probe, 2 U Taq DNA ligase (New England Biolabs, USA). The LDR parameters were as follows: 95°C for 2 min, 35 cycles at 94°C for 30 sec and 50°C for 2 min. Following the LDR reaction, 1 μl LDR reaction product was mixed with 1 μl ROX and 1 μl loading buffer. The mixture was then analyzed by the ABI Prism 377 DNA Sequencer (Applied Biosystems, USA). About 10% of the samples were randomly selected to perform the repeated assays and the results were 100% concordant.

### Statistical analysis

The statistical power analysis was performed using PS program (Power and Sample size calculations, Version 3.0.43) [34]. This study had more than 80% power to detect the differences between case and control subjects with an OR of more than 1.69 at a significant level of 0.05. All the three *SIRT1* tagSNPs were tested for confirmation with Hardy-Weinberg expectations by a goodness-of-fit χ2 test among the control subjects. Quantitative variables were expressed as mean ± standard deviation (SD), and qualitative variables were expressed as percentages. The differences of the demographic characteristics between the cases and controls were estimated using the χ2 test (for categorical variables) and student’s *t* test (for continuous variables). Association between the tagSNP and the risk for MI was evaluated using logistic regression analysis, adjusted by age, sex, body mass index (BMI), smoking, drinking, hypertension, diabetes and hyperlipidemia. The statistical analyses were performed using the SPSS software (version 21). The haplotype analysis on the polymorphisms was done using the SHEsis platform freely available online (http://202.120.7.14/analysis/myAnalysis.php) [33]. All the *P* values were corrected (*P*_*c*_) with the Bonferroni corrections and *P*_*c*_ < 0.05 was used as the criterion of statistical significance.

## Results

### Characteristics of the study population

The characteristics of the MI cases and controls were shown in Table 1. There was no statistically significant difference between cases and controls in terms of age. In the comparison of lipid profiles, serum total cholesterol (TC), triglycerides (TG), low density lipoprotein cholesterol (LDLC) were higher for patients than for controls (*P* < 0.001, *P* = 0.128, *P* < 0.001, respectively), whereas serum high density lipoprotein cholesterol (HDLC) levels were significantly higher among controls (*P* < 0.001). The average body mass index (BMI) and fasting plasma glucose (FPG) of the MI cases were significantly higher than that of the controls (*P* = 0.006 and *P* < 0.001, respectively). MI cases had higher levels of systolic blood pressure, diastolic blood pressure and there was also a higher prevalence of smokers, alcohol consumers, and individuals with hypertension, diabetes or hyperlipidemia among the patients. In addition, the number of female subjects in the cases was much lower than the male subjects. These data demonstrated that male gender, obesity, smoking, alcohol intake, hypertension, hyperlipidemia and diabetes mellitus were the important risk factors for developing MI in Chinese population.

**Table 1:** The characteristics of MI cases and controls.

Variable	Controls (n = 654)	Cases (n = 287)	*P* ^a^
Age (years)	61.37 ± 12.34	61.67 ± 11.95	0.728
Sex (male)	381 (58.3%)	223 (77.7%)	**< 0.001** ^b^
Smoking (%)	171 (26.1%)	173 (60.3%)	**< 0.001**
Drinking (%)	95 (14.5%)	80 (27.9%)	**< 0.001**
Hypertension (%)	229 (35.0%)	180 (62.7%)	**< 0.001**
Diabetes (%)	104 (15.9%)	136 (47.4%)	**< 0.001**
Hyperlipidemia (%)	245 (37.5%)	206 (71.8%)	**< 0.001**
BMI (kg/m^2^)	23.11 ± 1.87	23.49 ± 2.13	**0.006**
Systolic BP (mm Hg)	132.20 ± 18.89	140.24 ± 18.91	**< 0.001**
Diastolic BP (mm Hg)	72.73 ± 10.34	75.81 ± 11.48	**< 0.001**
FPG (mmol/L)	5.79 ± 1.90	6.63 ± 1.71	**< 0.001**
TG (mmol/L)	1.49 ± 0.82	2.07 ± 0.97	**< 0.001**
TC (mmol/L)	4.61 ± 1.14	4.74 ± 1.21	0.128
LDLC (mmol/L)	2.63 ± 0.91	3.04 ± 0.97	**< 0.001**
HDLC (mmol/L)	1.38 ± 0.67	1.19 ± 0.39	**< 0.001**

BMI, body mass index; FPG, fasting plasma glucose; TG, triglyceride; TC, total cholesterol; HDLC, high density lipoprotein cholesterol; LDLC, low density lipoprotein cholesterol.

^a^ Two-sided chi-square test or independent-samples *t*-test.

^b^ *P* values under 0.05 were indicated in bold font.

### Multivariate associations of *SIRT1* tagSNPs with the risk of MI

Three *SIRT1* tagSNPs (rs7069102, rs3818292 and rs4746720) were genotyped in 287 MI patients and 654 control subjects. The primary information for rs7069102, rs3818292 and rs4746720 polymorphisms was shown in S3 Table. Minor allele frequency (MAF) of all three tagSNPs in our controls was similar to MAF for Chinese in HapMap database (S3 Table). All the genotype frequency distributions of the three tagSNPs in our control subjects followed Hardy-Weinberg equilibrium proportions (all *P* values ≥ 0.30, S3 Table).

The allele and genotype distributions of the three tagSNPs in the cases and the controls were shown in Table 2. From the allelic association analysis, we found only rs7069102 showed statistical significance. The G allele frequency of rs7069102 in the MI patients was significantly higher than that in the control group (18.3% vs. 13.8%, Table 2). Unconditional logistic regression analysis revealed that G allele of rs7069102 had increased MI risk with odds ratio (OR) of 1.57 [95% confidence interval (CI) = 1.15–2.16, *P*_*c*_ = 0.015] after adjustment for conventional risk factors compared to C allele. Similarly, the combined CG/GG genotypes was associated with the increased MI risk (OR = 1.64, 95% CI = 1.14–2.35, *P*_*c*_ = 0.021) compared to the CC genotype. Taken together, our data indicated that *SIRT1* tagSNP rs7069102 may be associated with MI risk, and that individuals carrying G allele may have significantly increased MI susceptibility. However, we did not detect any association between rs3818292 or rs4746720 and the risk of MI in allelic or genotypic analyses (Table 2).

**Table 2:** Multivariate associations of the three tagSNPs in *SIRT1* gene with the risk of MI.

Type	Controls (n = 654) No. (%)	Cases (n = 287) No. (%)	OR (95% CI) ^a^	*P* ^a^	*P*_*c*_
***rs7069102***					
C	1128 (86.2)	469 (81.7)	1.00	-	
G	180 (13.8)	105 (18.3)	1.57 (1.15–2.16)	0.005	**0.015** ^**b**^
CC	486 (74.3)	192 (66.9)	1.00	-	
CG+GG	168 (25.7)	95 (33.1)	1.64 (1.14–2.35)	0.007	**0.021**
***rs3818292***					
A	942 (72.0)	418 (72.8)	1.00	-	
G	366 (28.0)	156 (27.2)	0.89 (0.69–1.16)	0.393	NS
GG	56 (8.6)	27 (9.4)	1.00	-	
AG+AA	598 (91.4)	260 (90.6)	0.98 (0.54–1.77)	0.932	NS
***rs4746720***					
C	564 (43.1)	242 (42.2)	1.00	-	
T	744 (56.9)	332 (57.8)	1.11 (0.88–1.42)	0.383	NS
CC	119 (18.2)	46 (16.0)	1.00	-	
CT+TT	535 (81.8)	241 (84.0)	1.20 (0.77–1.85)	0.419	NS

*P*_*c*_, Bonferroni corrected *P*; NS, not significant.

^a^ Adjusted for age, sex, BMI, smoking, drinking, hypertension, diabetes and hyperlipidemia.

^b^ *P*_*c*_ values under 0.05 were indicated in bold font.

### Stratification analyses of *SIRT1* rs7069102 polymorphism and risk of MI

We further evaluated the genotypes and MI susceptibility after stratifying the subjects by age, sex, status of smoking or drinking, and MI-associated phenotypes including hypertension, diabetes or hyperlipidemia. When stratification by age was performed, the increased risk of MI was more evident among younger subjects (≤ 55 years old, N = 307) carrying G allele (OR = 2.21, 95% CI = 1.13–4.31, *P*_*c*_ = 0.040, Table 3) or the combined CG/GG genotype (OR = 2.27, 95% CI = 1.13–4.55, *P*_*c*_ = 0.042, Table 3), while there was no significant association in the group older than 55 years old (N = 634). However, no more evident association between rs7069102 polymorphism and MI risk was observed among subgroups by sex, status of smoking or drinking, etc (data not shown).

**Table 3:** Multivariate associations of the rs7069102 in *SIRT1* gene with the risk of MI by further stratification for age.

Type	Controls No. (%)	Cases No. (%)	OR (95% CI) ^a^	*P* ^a^	*P*_*c*_
**≤55**	**n = 214**	**n = 93**			
C	381 (89.0)	152 (81.7)	1.00	-	
G	47 (11.0)	34 (18.3)	2.21 (1.13–4.31)	0.020	**0.040** ^**b**^
CC	168 (78.5)	60 (64.5)	1.00	-	
CG+GG	46 (21.5)	33 (35.5)	2.27 (1.13–4.55)	0.021	**0.042**
>**55**	**n = 440**	**n = 194**			
C	747 (84.9)	317 (81.7)	1.00	-	
G	133 (15.1)	71 (18.3)	1.32 (0.91–1.90)	0.141	NS
CC	318 (72.3)	132 (68.0)	1.00	-	
CG+GG	122 (27.7)	62 (32.0)	1.33 (0.87–2.05)	0.192	NS

*P*_*c*_, Bonferroni corrected *P*; NS, not significant.

^a^ Adjusted for sex, BMI, smoking, drinking, hypertension, diabetes and hyperlipidemia.

^b^ *P*_*c*_ values under 0.05 were indicated in bold font.

### Association between the haplotypes of *SIRT1* tagSNPs with the risk of MI

As shown in Figure B in S1 Fig., all the three tagSNPs were located in one haplotypic block. We thus further compared the haplotype frequencies of the three tagSNPs between MI group and controls. Four common haplotypes (frequency > 3%) derived from the three tagSNPs accounted for almost 100% of the haplotype variations. Among the four common haplotypes, only the haplotype rs7069102G-rs3818292A-rs4746720T carrying G allele of rs7069102 was found to be associated with an increased risk for MI (OR = 1.41, 95% CI = 1.09–1.84, *P*_*c*_ = 0.040, Table 4). Furthermore, the increased risk of MI was more pronounced among younger subjects (≤55 years old) carrying haplotype rs7069102G-rs3818292A-rs4746720T, although it was only borderline significant (OR = 1.81, 95% CI = 1.12–2.93, *P*_*c*_ = 0.056, Table 5).

**Table 4:** Association between haplotypes of the three tagSNPs in *SIRT1* gene with the risk of MI.

Haplotype^a^	Controls (n = 654) No. (%)	Cases (n = 287) No. (%)	OR (95% CI)	*P*	*P*_*c*_
C A C	562.99 (43.0)	240.87 (42.0)	0.96 (0.79–1.17)	0.678	NS
C A T	200.01 (15.3)	72.13 (12.6)	0.80 (0.60–1.07)	0.124	NS
C G T	364.99 (27.9)	154.87 (27.0)	0.96 (0.77–1.19)	0.691	NS
G A T	179.00 (13.7)	105.00 (18.3)	1.41 (1.09–1.84)	0.010	**0.040** ^b^

*P*_*c*_, Bonferroni corrected *P*; NS, not significant.

^*a*^ The allelic sequence in the haplotypes is in the following order: rs7069102, rs3818292, rs4746720.

^b^ *P*_*c*_ values under 0.05 were indicated in bold font.

**Table 5:** Association between haplotypes of the three tagSNPs in *SIRT1* gene with the risk of MI among younger subjects (≤ 55 years old).

Haplotype^a^	Controls (n = 214) No. (%)	Cases (n = 93) No. (%)	OR (95% CI)	*P*	*P*_*c*_
C A C	202.00 (47.2)	78.00 (41.9)	0.81 (0.57–1.14)	0.229	NS
C A T	55.00 (12.9)	29.00 (15.6)	1.25 (0.77–2.04)	0.364	NS
C G T	124.00 (29.0)	45.00 (24.2)	0.78 (0.53–1.16)	0.223	NS
G A T	47.00 (11.0)	34.00 (18.3)	1.81 (1.12–2.93)	0.014	0.056

*P*_*c*_, Bonferroni corrected *P*; NS, not significant.

^*a*^ The allelic sequence in the haplotypes is in the following order: rs7069102, rs3818292, rs4746720.

## Discussion

MI is a complex multifactorial, polygenic disorder which results from the interaction between individual’s genetic makeup and various environmental factors. The principal pathogenesis of MI is the rupture of coronary atherosclerotic plaques. Recent studies have demonstrated the protective roles of *SIRT*1 in inflammation processes, vascular endothelial homeostasis and atherosclerosis [20,35,36], providing evidence that *SIRT1* may play an important role in the pathogenesis of MI. However, the association between SNPs in *SIRT1* gene and MI risk is still largely unknown. In the present study, we performed a genetic association analysis on the three *SIRT1* tagSNPs (rs7069102, rs3818292 and rs4746720) in 287 MI patients and 654 controls. Our result showed that *SIRT1* rs7069102 G allele is associated with a significantly increased risk of MI. We did not detect any association between rs3818292 and the risk of MI in allelic or genotypic analyses, which was in line with the previous report of rs3740051 (captured by rs3818292, r^2^ = 1.0, S1 Table) in a Chinese population [37]. The haplotype (rs7069102G-rs3818292A-rs4746720T) containing the rs7069102 G allele also confers increased risk of MI. Further stratified analyses revealed that the increased risk of MI was more evident among younger subjects in allelic, genotypic or haplotypic analyses, but not among older subjects. The potential risk of MI in older individuals is more likely due to the aging effect (e.g., weak immune system, relative high level exposure to environmental risk factors) rather than direct genetic effects. Thus, the *SIRT1* rs7069102 polymorphism might be more influential among younger subjects. These findings support our prior speculation that the *SIRT1* polymorphism may contribute to susceptibility to MI. To the best of our knowledge, this is the first study showing that the tagSNP rs7069102 and haplotype rs7069102G-rs3818292A-rs4746720T in *SIRT1* gene are associated with an increased risk of MI in Chinese subjects.

Though SNPs in *SIRT1* gene have been widely studied in other traits (S1 Table), there is no published paper reporting the direct effects of these three tagSNPs on MI risk [38–50]. An association study of *SIRT1* gene variations with visceral obesity found that the rs7069102 C allele was associated with reduced risk of obesity in Belgian Caucasians [40]. In Japanese population, Yasuhiko *et al*. found that the serum levels of TC and LDLC were significantly higher in G allele carriers of rs7069102 compared with CC genotype in male hemodialysis patients [41]. They also found that G allele of rs7069102 carried a high risk not only for obesity in men but also for hypertension [42]. Our data showed that individuals carrying G allele may have significantly increased MI susceptibility. However, neither lipid levels nor BMI was associated with rs7069102 genotypes in the present study (S2 Fig.). Therefore, we speculate that SIRT1 rs7069102 polymorphism exert its effect on MI development independent of individual’s lipid metabolism. Further functional studies on this polymorphism are needed to elucidate the underlying molecular mechanisms of the observed association.

Besides, rs7069102 is a tagSNP which means that it captures the genetic information of other closely linked SNPs (high LD), effectively enabling us to reduce the number of markers needed to analyze the whole gene. Thus, we should keep in mind that the association of the rs7069102 polymorphism with the risk of MI may be due to a direct causative effect of this SNP, or because it is in LD with other functional variants located in or near the *SIRT1* gene and is associated with the risk of MI. Therefore, further extensive analyses for this locus, dense LD mapping or further confirmation studies are also required to link the *SIRT1* locus to the genetic susceptibility of MI as a whole.

Several limitations need to be addressed in this case-control study. First, the patients and controls were enrolled from hospitals and may not represent the general population. Nonetheless, the genotype distribution of the controls was in Hardy-Weinberg equilibrium. Second, although the statistical power (67.5% in all subjects and 66.0% in younger subjects) of rs7069102 is not very low, more samples should be recruited to improve the statistical power of our analysis. Third, the tagSNPs we selected are non-coding, and we therefore assume these variants to be linked with one or more functional variants within the *SIRT1* gene or its regulatory regions. Future fine mapping of the *SIRT1* gene may detect such functional variants. Finally, given that the results of the present study were not replicated, further studies in different population could help to validated the significance of the association between these tagSNPs and the risk of MI. However, our observations provided valuable insights and interesting information and might serve to guide future studies in this area.

In summary, our study provides the first evidence that the G allele of *SIRT1* tagSNP rs7069102 and the haplotype (rs7069102G-rs3818292A-rs4746720T) are associated with an increased risk of MI. Our finding suggests that the genetic polymorphism within *SIRT1* gene may play a role in the occurrence of MI in a Chinese population, although further studies with larger sample size and in diverse ethnic populations are required to confirm the general validity of our findings.

## Supporting Information

S1 Fig Schematic of *SIRT1* gene structure and pairwise LD between the three tagSNPs. S1A Fig. provides the details of the *SIRT1* gene structure. *SIRT1* gene is composed of 9 exons and spans 33.72kb. The exons are represented as dark gray boxes. D’ values are plotted as a graph to show linkage disequilibrium between the three tagSNPs in S1B Fig. Details of the picked tagSNPs and respective alleles captured are also provided in S1B Fig.
(TIF)

S2 Fig The association of rs7069102 genotypes with BMI (A) and lipid levels (B). (TIF)

S1 Table The information for alleles captured by rs7069102, rs3818292 and rs4746720. (DOC)

S2 Table The sequences of the primers and probes used to genotype the rs7069102, rs3818292 and rs4746720 polymorphisms. (DOC)

S3 Table Primary information for rs7069102, rs3818292 and rs4746720 polymorphisms. (DOC)
