# Supplementary figures and images for: A TagSNP in SIRT1 Gene Confers Susceptibility to Myocardial Infarction in a Chinese Han Population
Source: PLoS One. 2015 Feb 23;10(2):e0115339. doi: 10.1371/journal.pone.0115339 (PMC4338141; doi:10.1371/journal.pone.0115339)

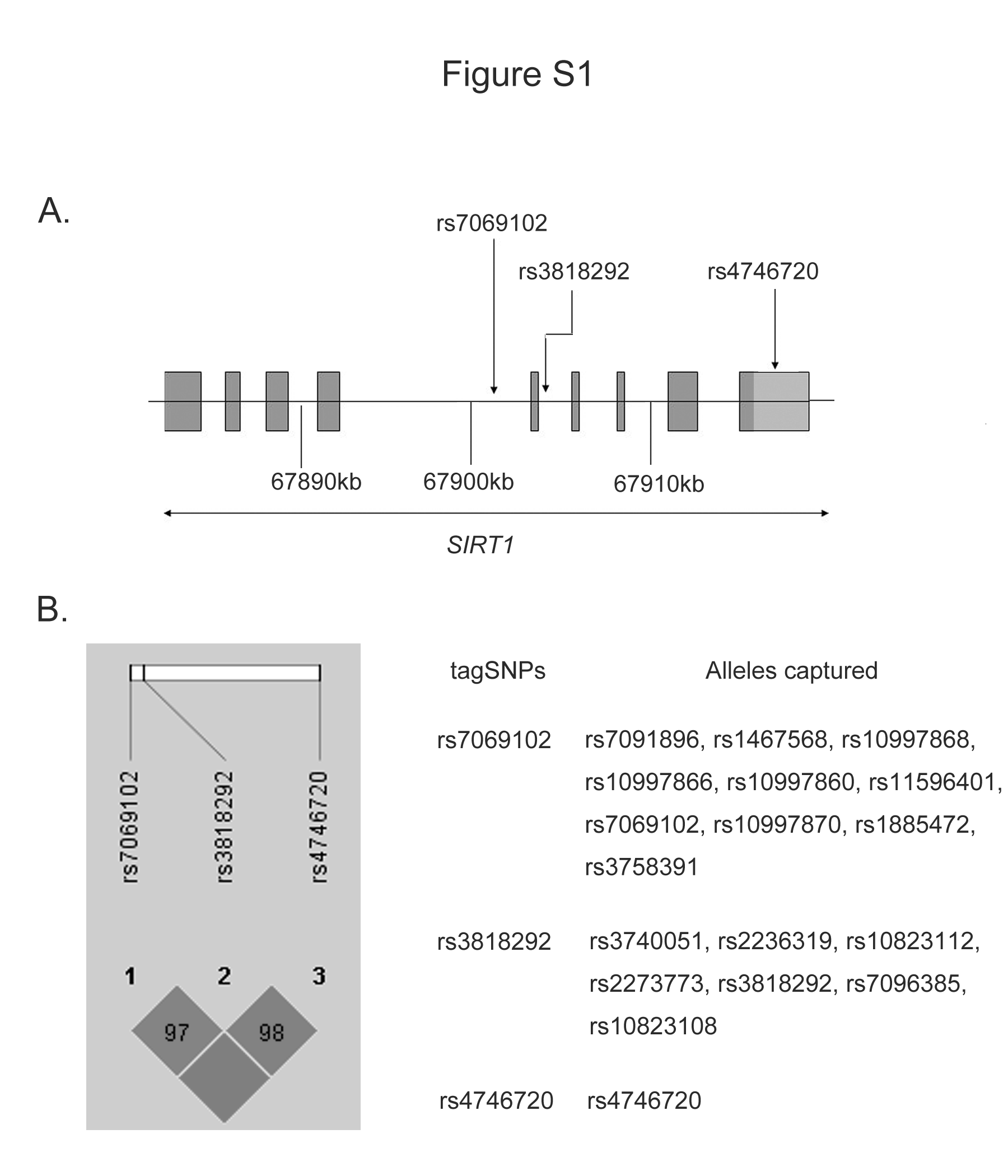

Supplement: S1 Fig — S1A Fig. provides the details of the SIRT1 gene structure. SIRT1 gene is composed of 9 exons and spans 33.72kb. The exons are represented as dark gray boxes. D’ values are plotted as a graph to show linkage disequilibrium between the three tagSNPs in S1B Fig. Details of the picked tagSNPs and respective alleles captured are also provided in S1B Fig. (TIF) [file pone.0115339.s001.tif]

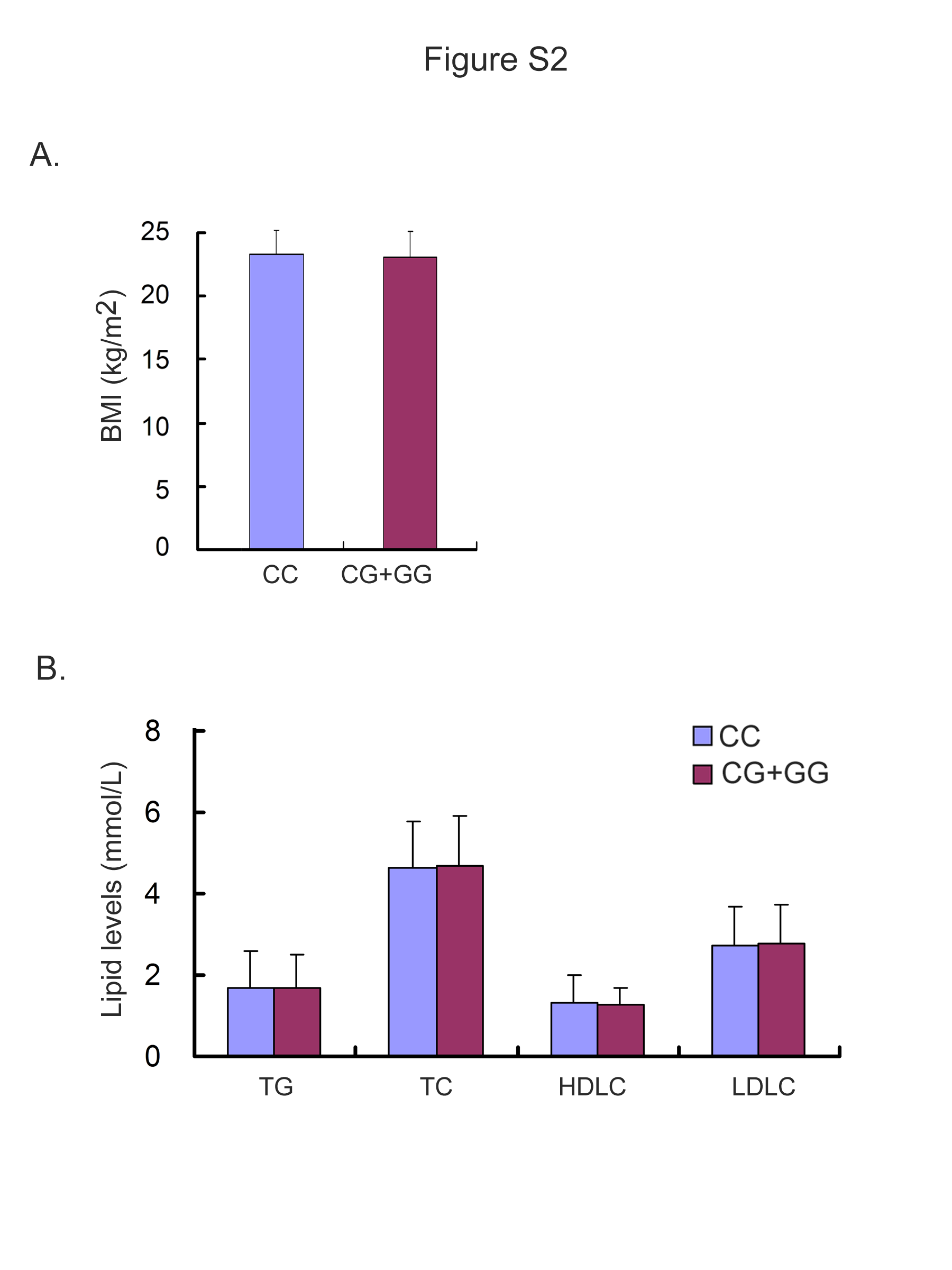

Supplement: S2 Fig — (TIF) [file pone.0115339.s002.tif]
