# Supplementary material for: A TagSNP in SIRT1 Gene Confers Susceptibility to Myocardial Infarction in a Chinese Han Population
Source: PLoS One. 2015 Feb 23;10(2):e0115339. doi: 10.1371/journal.pone.0115339 (PMC4338141; doi:10.1371/journal.pone.0115339)
Supplement: S1 Table — (DOC) [file pone.0115339.s003.doc]

**Table S1.**The information for alleles captured by rs7069102, rs3818292 and rs4746720.

| **Alleles captured** | **Location** | **tagSNP** | **r^2^** | **Potential function** ^a^ | | **Reported traits** | **Reference** |
| --- | --- | --- | --- | --- | --- | --- | --- |
|  |  |  |  | **TFBS** | **miRNA** |  |  |
| rs7091896 | intron 8 | rs7069102 | 1.0 | - | - | - | - |
| rs1467568 | intron 8 | rs7069102 | 1.0 | - | - | BMI | [38,39] |
| rs10997868 | intron 4 | rs7069102 | 0.916 | - | - |  |  |
| rs10997866 | intron 4 | rs7069102 | 1.0 | - | - | - | - |
| rs10997860 | intron 1 | rs7069102 | 1.0 | - | - | - | - |
| rs11596401 | intron 4 | rs7069102 | 1.0 | - | - | - | - |
| rs7069102 | intron 4 | rs7069102 | 1.0 | - | - | Visceral obesity, serum lipid levels and human lifespan | [40-44] |
| rs10997870 | intron 6 | rs7069102 | 0.916 | - | - | - | - |
| rs1885472 | intron 4 | rs7069102 | 1.0 | - | - | - | - |
| rs3758391 | upstream 2kb | rs7069102 | 1.0 | Y | - | Aging and cognitive functioning | [45,46] |
| rs3740051 | upstream 2kb | rs3818292 | 1.0 | Y | - | Myocardial infarction and diabetic nephropathy | [37,47] |
| rs2236319 | intron 3 | rs3818292 | 0.932 | - | - | - | - |
| rs10823112 | intron 7 | rs3818292 | 1.0 | - | - | - | - |
| rs2273773 | exon 5 | rs3818292 | 1.0 | - | - | Obesity, cholesterol metabolism, human lifespan, diabetic nephropathy, psychosis and Alzheimer's disease | [41-44,47-49] |
| rs3818292 | intron 5 | rs3818292 | 1.0 | - | - | Serum lipid levels, visceral obesity and diabetic nephropathy | [40,43,47] |
| rs7096385 | intron 4 | rs3818292 | 1.0 | - | - | - | - |
| rs10823108 | intron 4 | rs3818292 | 1.0 | - | - | Human lifespan and diabetic nephropathy | [44,47] |
| rs4746720 | 3' UTR | rs4746720 | 1.0 | - | Y | Aging, diabetic nephropathy and longevity | [45,47,50] |

^a^ The potential functions of the SNPs were predicted by online webserver (http://snpinfo. niehs.nih.gov/snpinfo/snpfunc.htm).
