# Supplementary material for: A TagSNP in SIRT1 Gene Confers Susceptibility to Myocardial Infarction in a Chinese Han Population
Source: PLoS One. 2015 Feb 23;10(2):e0115339. doi: 10.1371/journal.pone.0115339 (PMC4338141; doi:10.1371/journal.pone.0115339)
Supplement: S2 Table — (DOC) [file pone.0115339.s004.doc]

**Table S2.** The sequences of the primers and probes used to genotype the rs7069102, rs3818292 and rs4746720 polymorphisms.

| **Name** | **Sequence (5’-3’)** |
| --- | --- |
| **Primers** |  |
| rs7069102-F | CCATGCCCAGCTTTCTTAGT |
| rs7069102-R | CCAGGAGGTGGAGCTTGTAG |
| rs3818292-F | ACTGCCATCGAGAAGTGGAG |
| rs3818292-R | CACTGCACTCAGCCAGAAAA |
| rs4746720-F | CAAAAAGCCATCGGAATGTT |
| rs4746720-R | CCCCACATATTGTTGACTTCC |
| **Probes** |  |
| rs7069102-FAM | P-CAGAGATTATGCCTTTCTTTTTTTTTTTTTTTTTTTTTTTT-FAM |
| rs7069102-C | TTTTTTTTTTTTTTTTTTCTGCAGAAATAATGGCTTTTCTG |
| rs7069102-G | TTTTTTTTTTTTTTTTTTTTCTGCAGAAATAATGGCTTTTCTC |
| rs3818292-FAM | P-ATCTGAGGTTTTCTATAATTTTTTTTTTTTTTTTTTTTTTTTTT-FAM |
| rs3818292-A | TTTTTTTTTTTTTTTTTTTTTTTGAAATCAAAAGCAAATTAAT |
| rs3818292-G | TTTTTTTTTTTTTTTTTTTTTTTTTGAAATCAAAAGCAAATTAAC |
| rs4746720-FAM | P-AAGTTTAGCGTAACAGATTTTTTTTTTTTTTTTTTTTTTTTTTTTTTTTT-FAM |
| rs4746720-C | TTTTTTTTTTTTTTTTTTTTTTTTAAATAATTGTGTTAAAGAATCAG |
| rs4746720-T | TTTTTTTTTTTTTTTTTTTTTTTTTTAAATAATTGTGTTAAAGAATCAA |
