# Supplementary material for: A TagSNP in SIRT1 Gene Confers Susceptibility to Myocardial Infarction in a Chinese Han Population
Source: PLoS One. 2015 Feb 23;10(2):e0115339. doi: 10.1371/journal.pone.0115339 (PMC4338141; doi:10.1371/journal.pone.0115339)
Supplement: S3 Table — (DOC) [file pone.0115339.s005.doc]

**Table S3.** Primary information for rs7069102, rs3818292 and rs4746720 polymorphisms.

| **Genotyped SNPs** | **rs7069102** | **rs3818292** | **rs4746720** |
| --- | --- | --- | --- |
| Chr Pos (Genome Build 104.0) | 69663120 | 69666901 | 69676830 |
| Pos in *SIRT1* gene | Intron 4 | Intron 5 | 3' UTR |
| MAF for Chinese(CHB) in HapMap^a^ | 0.144 | 0.314 | 0.407 |
| MAF in our controls (n = 654) | 0.138 | 0.280 | 0.431 |
| *P* value for HWE test in our controls^b^ | 0.899 | 0.352 | 0.679 |

^a^ MAF: minor allele frequency.

^b^ HWE: Hardy-Weinberg equilibrium.
